# Severe COVID-19 Pneumonia Treated by Intensive Immune Suppression Therapy With a Combination of Steroid Pulse and Tocilizumab Followed by a Tapering Dose of Steroid Therapy During the Delta (B.1.617.2) Variant Outbreak: A Successfully Treated Case

**DOI:** 10.7759/cureus.19340

**Published:** 2021-11-07

**Authors:** Ken-ichi Muramatsu, Kouhei Ishikawa, Arisa Komatsu, Kei Jitsuiki, Youichi Yanagawa

**Affiliations:** 1 Acute Critical Care Medicine, Shizuoka Hospital, Juntendo University, Izunokuni, JPN; 2 Respiratory Medicine, Shizuoka Hospital, Juntendo University, Izunokuni, JPN

**Keywords:** organizing pneumonia, tocilizumab, steroid pulse, delta variant, covid-19

## Abstract

A 55-year-old man developed a low-grade fever (day 1). His wife had already been infected with COVID-19 four days previously and he had been isolated in his house as a close contact. Polymerase chain reaction for COVID-19 was positive. He had untreated diabetes mellitus. On day 7, his percutaneous saturated oxygen fell to <70% and he was transported to a hospital by ambulance. He underwent tracheal intubation, mechanical ventilation, and treatments with half steroid pulse, tocilizumab, remdesivir, and heparin. However, his ratio of arterial oxygen partial pressure to fractional inspired oxygen (P/F ratio) decreased to 120 under mechanical ventilation and he was transported to our hospital. On arrival, he did not synchronize with mechanical ventilation well. Initially, he was treated using a muscle relaxant and deep sedation to facilitate complete synchronization with mechanical ventilation and his P/F ratio improved to 247; thus, he was treated with mechanical ventilation alone with intermittent placement in the prone position. In addition, he was treated with steroid pulse therapy after steroid tapering therapy for nearly one month, glycyrrhizin, γ-globulin, azithromycin, and heparin. On day 20, the tracheal tube was removed after the improvement of the P/F ratio. We herein present the case of a patient with severe COVID-19 pneumonia who survived following treatment by intensive immune suppression therapy, including the combination of steroid pulse and tocilizumab, followed by a tapering dose of steroid therapy, after an outbreak of COVID-19 Delta variant. Further studies are needed to investigate the usefulness of this regimen.

## Introduction

The following medical treatment for COVID-19 has received insurance coverage in Japan (October, 2021): 1) one-time drip infusion of neutralizing antibodies (casirivimab/imdevimab or sotrovimab) for patients with mild or moderate illness; 2) dexamethasone (6 mg per day for 10 days) as an oral medicine for patients requiring oxygen; 3) drip infusion of remdesivir (200 mg on day 1, 100 mg on days 2-5) for patients with pneumonia, percutaneous saturated oxygen <94%, requiring oxygen or requiring mechanical ventilation; and 4) baricitinib (an oral Janus kinase inhibitor: 4 mg per day for 14 days) for patients with moderate or severe illness. Recently, tocilizumab (an interleukin-6 inhibitor) has been approved for use by the Ministry of Health, Labor and Welfare, Japan for patients requiring oxygen, mechanical ventilation, or vasopressor.

In our department, in addition to the use of steroids, remdesivir and heparin, unspecific antiviral therapies and immune modulation therapy using intravenous immunoglobulin (IVIG), glycyrrhizin, and macrolide antibiotics have been applied for the treatment of COVID-19 [1]. However, after the start of an outbreak of COVID-19 Delta variant infections in Japan, this treatment failed to improve survival. Accordingly, we developed a new regimen with a combination of steroid pulse and tocilizumab followed by a tapering dose of steroids for nearly one month. This approach to the treatment of cryptogenic organizing pneumonia induced by COVID-19 provided intense immune suppression.

## Case presentation

A 55-year-old man developed a low-grade fever (day 1). His wife had had already been infected by COVID-19 four days previously and he had been isolated in his house as a close contact. Polymerase chain reaction for COVID-19 became positive. He had diabetes mellitus without treatments. His son was also infected with COVID-19 at this time. On day 6, his percutaneous saturated oxygen became <90%. The next day, his percutaneous saturated oxygen (SPO2) fell to <70% and he was transported to a hospital by ambulance. He underwent tracheal intubation, mechanical ventilation, and treatments with half steroid pulse (500 mg of methylprednisolone per day × three days), drip infusion of tocilizumab (480 mg, day 1), drip infusion of remdesivir (200 mg, day 1; 100 mg, days 2 and 3) and continuous drip infusion of heparin. However, his ratio of arterial oxygen partial pressure (PaO2 in mmHg) to fractional inspired oxygen (FiO2 expressed as a fraction) (P/F ratio) decreased to 120 under mechanical ventilation with FiO2, 0.7; pressure support (PS), 20 cmH2O; positive end-expiratory pressure (PEEP), 15 cmH2O. His arterial pressure also dropped, which necessitated vasopressor treatment. He was transported to our hospital to apply veno-venous extracorporeal membrane oxygenation (VV-ECMO) on day 9.

**Figure 1:**
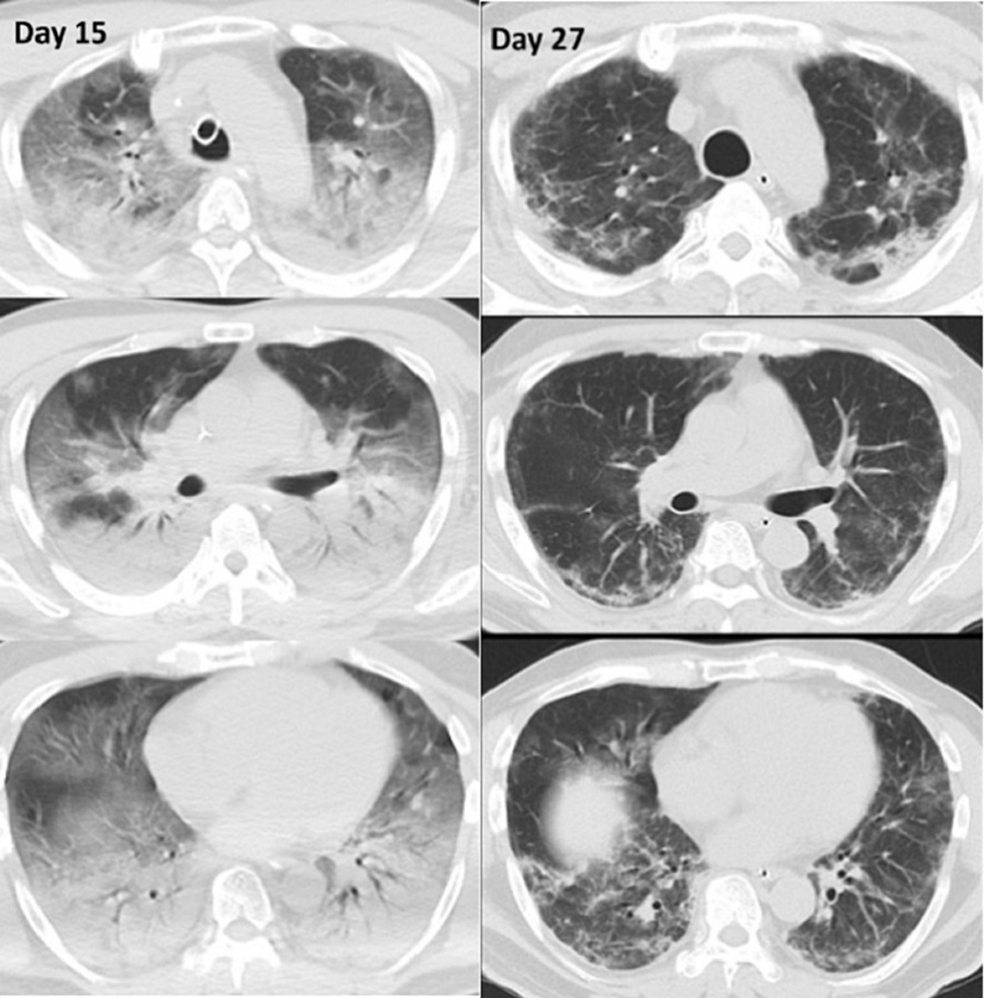
Chest CT on days 15 (left) and 27 (right). CT on day 15 demonstrates bilateral dorsal atelectasis and ventral ground glass opacities. CT on day 27 shows multiple organized lesions in the bilateral lungs.

On arrival, his vital signs were as follows: Glasgow Coma Scale, E1VTM1 under sedation; blood pressure, 147/92 mmHg; heart rate, 110 beats per minute; respiratory rate, 24 breaths per minute; and body temperature, 36.2°C. He did not synchronize with mechanical ventilation well. Chest CT revealed bilateral dorsal atelectasis and a ventral ground glass appearance (Figure 1).

Electrocardiography showed sinus tachycardia. The results of the biochemical analysis are shown in Table 1.

**Table 1:** Results of a biochemical analysis.

Variables	Level
White blood cell count	8600 /μL
Hemoglobin	13.8 g/dL
Platelet	18.7 × 104/μL
Total protein	5.7 g/dL
Albumin	2.7 g/dL
Aspartate aminotransferase	47 U/L
Alanine aminotransferase	44 U/L
Glutamyl transpeptidase	186 IU/L
Creatinine phosphokinase	65 U/L
Amylase	135 U/L
Glucose	283 mg/dL
Hemoglobin A1c	9.60%
Blood urea nitrogen	29.9 mg/dL
Creatinine	1.23 mg/dL
Sodium	143 mEq/L
Potassium	4.7 mEq/L
Chloride	108 mEq/L
C-reactive protein	3.51 mg/dL
Alkaline phosphatase	100 IU/L
Lactate dehydrogenase	623 IU/L
Krebs von den Lungen-6 (KL-6)	1135.3 U/mL
Ferritin	2377 ng/mL
Prothrombin time international normalized ratio	1.08
Activated partial thromboplastin time	28.6 s
D-dimer	17.5 μg/mL

**Figure 2:**
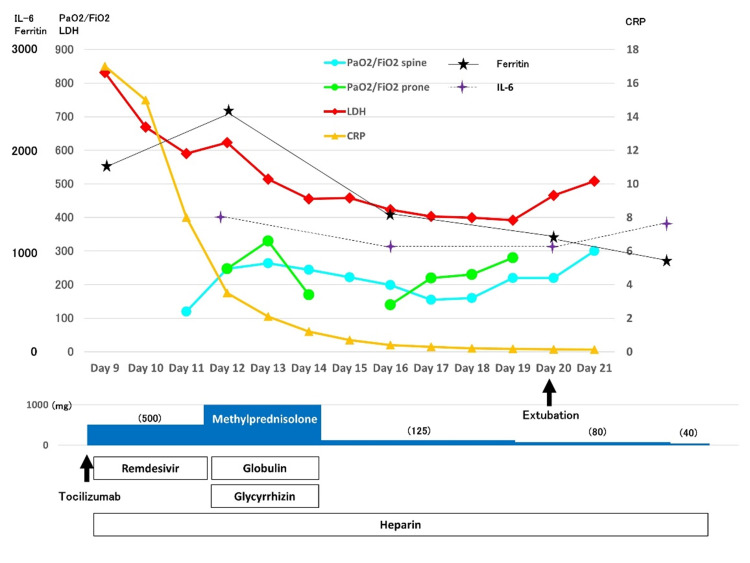
The time course of intensive treatment. After steroid pulse following steroid tapering therapy, the P/F ratio decreased until day 17 and then increased. On day 20, the tracheal tube was removed. P/F, ratio of arterial oxygen partial pressure to fractional inspired oxygen; LDH, lactate dehydrogenase; KL-6, Krebs von den Lungen-6.

Initially, he was treated using muscle relaxant and deep sedation to allow him to completely synchronize with mechanical ventilation, whether VV-ECMO was indicated or not. A blood gas analysis (under FiO2, 0.8; PEEP, 15 cmH2O; PS, 20 cmH2O) revealed pH, 7.226; PO2, 198 mmHg; PCO2, 66 mmHg; HCO3-, 25.1 mmol/L, base excess -2.4 mmol/L, and his P/F ratio was 247; thus, he was treated with mechanical ventilation alone using a low tidal and high PEEP strategy with intermittent placement in the prone position. In addition, he was treated with steroid pulse (methylprednisolone 1000 mg × three days) following steroid tapering therapy for a month, glycyrrhizin (20 mL × three days), γ-globulin (5 g × three days), azithromycin (500 mg × three days), tazobactam/piperacillin (13.5 g per day), and continuous heparin infusion. Our recent standard regimen includes the combination of tocilizumab and steroid pulse; however, tocilizumab had already been infused in the previous hospital; thus, that infusion of tocilizumab was canceled. The time course of treatment, P/F ratio, and main results of the biochemical analysis are shown in Figure 2.

After steroid pulse following steroid tapering therapy, the P/F ratio decreased until day 17 and then increased. On day 20, the tracheal tube was removed. He showed transient delirium, hallucination, and weakness of muscle strength of all extremities due to disuse atrophy and/or critical illness myopathy. On day 25, his percutaneous saturation improved to approximately 95% under room air while on bed rest, and was transferred to a general ward. Chest CT on day 27 showed multiple organized lesions in the bilateral lungs and a respiratory physiological function test showed both restrictive and obstructive ventilatory disturbance [vital capacity, 1.8 L (49%); forced expiratory volume in one second, 1.4 L (47%)]. From day 29, methylprednisolone infusion (40 mg per day) was switched to 20 mg per day orally. Oral steroid medication was tapered every five days and finally withdrawn on day 32. He was discharged on day 37 still with hypoxia (SPO2, 86%; heart rate, 144 beats per minute under the six-minute walk test) on effort.

## Discussion

Since the outbreak of the Delta variant, COVID-19 has become more transmissible and is associated with higher rates of hospitalization, and the severity has significantly worsened [2]. Following the outbreak of the COVID-19 Delta variant in Japan, multimodal therapy, including immune modulation, in our department failed to improve survival. 

COVID-19 infection with immunologic complications, including secondary hemophagocytic lymphohistiocytosis, results in cytokine storm syndrome and acute respiratory distress syndrome [3]. Viral features, decreased levels of interferons, increased neutrophil extracellular traps, increased pyroptosis, various genetic mutations, and possibly other unknown mechanisms are risk factors for a severe disease course and the occurrence of cytokine storm in cases of COVID-19 [3]. Once immunologic complications like cytokine storm occur, anti-viral treatment alone is not sufficient and should be combined with appropriate anti-inflammatory treatment [3]. Cytokine storms are triggered by the action of interferon-γ, tumor necrosis factor-α, interleukin (IL)-1, IL-2, and IL-6 [4]. Among the cytokines, the IL-6 levels are significantly elevated and associated with adverse clinical outcomes in COVID-19 patients [4]. Higher levels of IL-6 among patients with COVID-19 have been shown to be associated with more severe complications, higher rates of acute respiratory distress syndrome requiring intensive care, and mortality [4]. Inhibition of IL-6 may be a novel therapeutic target for the management of dysregulated host responses in COVID-19 patients [4]. However, randomized controlled trials have reported no significant effect of tocilizumab monotherapy against COVID-19 [5], possibly due to IL-6 pathway inhibition along being insufficient management in critical cases. Accordingly, we recently adapted the combination of steroid pulse and tocilizumab based on previous reports, after the previous regimen failed to improve survival [5-6]. The mortality rate was significantly lower with combined therapy of steroid pulse and tocilizumab than in the control group, which itself had a lower mortality rate than the groups receiving steroid pulse or tocilizumab alone [5-6]. Combining tocilizumab and steroid pulse therapy to quickly achieve strong immunosuppression was considered better than either alone. Among patients with severe COVID-19 pneumonia, CT imaging along with postmortem lung biopsy and autopsy indicate that the majority of patients have secondary acute fibrinous and organizing pneumonia, which results in progression to advanced lung fibrosis or post-COVID interstitial lung disease [7-8]. In the case of fibrinous and organizing pneumonia, long-term steroid treatment might be required to obtain disease remission [9]. Accordingly, we adapted steroid tapering therapy after steroid pulse. The present case occurred after the Delta variant outbreak in Japan and was unable to achieve complete recovery from COVID-19 pneumonia by a combination of steroid half-pulse and tocilizumab alone, so additional steroid pulse with long-term tapering of steroid therapy had to be added to control the patient’s COVID-19 pneumonia. This patient survived with these treatments plus prone positioning. However, even this intensive immune suppression therapy failed to achieve complete recovery of the pulmonary function.

A prone position is known to improve mortality in patients with acute respiratory distress syndrome. The prone position leads to a relief of severe hypoxemia due to a reduction in the overinflated lung area, promotion of alveolar recruitment, and reduction in ventilation/perfusion mismatch [10]. Indeed, death was reportedly decreased by up to one-third in hospitalized patients with severe respiratory complications, even those with COVID-19 [10]. However, trained and qualified nursing and respiratory therapy staff are crucial for obtaining successful results with this approach, as severe life-threatening events may occur at any given time (self-extubation, hemodynamic instability, lack of adequate sedation, pressure ulcers, etc.) [10]. Nevertheless, the present case supports the benefits of prone positioning respiratory therapy when performed by skilful staff.

One limitation of this case report is that the present case was initially treated with combined steroids (half-pulse) and tocilizumab, but his condition still deteriorated. Subsequently, the patient was transferred and treated with a combination of pulse-dose steroids, antibiotics (piperacillin-tazobactam and azithromycin), glycyrrhizin, gamma-globulin, muscle-relaxant, and intermittent prone ventilation. There are thus a number of confounders that may have affected the patient's disease condition, leaving us unable to tell for sure what helped improve the disease condition in our patient. This patient was also at a high risk of developing concomitant bacterial pneumonia after receiving steroids and tocilizumab during initial hospitalization, and broad-spectrum antibiotics, which the patient received the following transfer to another facility, might have helped his disease course. Furthermore, this was a case report; therefore, the validity of the combination of steroid pulse and tocilizumab followed by a tapering dose of steroid therapy should be evaluated following the accumulation of further cases or in a randomized controlled trial in the future.

## Conclusions

In our department, in addition to the use of steroids, remdesivir and heparin, unspecific antiviral therapies and immune modulation therapy using intravenous immunoglobulin (IVIG), glycyrrhizin, and macrolide antibiotics have been applied for the treatment of COVID-19. However, following the outbreak of COVID-19 Delta variant infections in Japan, this treatment failed to improve survival.

We presented a case of severe COVID-19 pneumonia in which survival was obtained following a new regimen with intensive immune suppression therapy, including the combination of tocilizumab and steroid pulse after steroid tapering therapy following the COVID-19 Delta variant outbreak. The regimen in the present case might have helped our patient, but we cannot draw firm conclusions based on a single case. Further studies are needed to determine the usefulness of this regimen.

## References

[1] Jitsuiki K, Katayama I, Iida T, Nagatomo S, Yanagawa Y (2020). Successful treatment of elderly male with COVID-19 infection with severe acute respiratory distress syndrome using multimodal therapy, including immune modulation therapy. Cureus.

[2] Shah SA, Moore E, Robertson C (2021). Predicted COVID-19 positive cases, hospitalisations, and deaths associated with the Delta variant of concern, June-July, 2021. Lancet Digit Health.

[3] Soy M, Keser G, Atagündüz P, Tabak F, Atagündüz I, Kayhan S (2020). Cytokine storm in COVID-19: pathogenesis and overview of anti-inflammatory agents used in treatment. Clin Rheumatol.

[4] Coomes EA, Haghbayan H (2020). Interleukin-6 in Covid-19: A systematic review and meta-analysis. Rev Med Virol.

[5] Toda M, Fujii K, Yoshifuji A (2021). Clinical efficacy and safety of combination therapy of tocilizumab and steroid pulse therapy for critical COVID-19 in HD patients. Clin Exp Nephrol.

[6] Van den Eynde E, Gasch O, Oliva JC (2021). Corticosteroids and tocilizumab reduce in-hospital mortality in severe COVID-19 pneumonia: a retrospective study in a Spanish hospital. Infect Dis (Lond).

[7] Kory P, Kanne JP (2020). SARS-CoV-2 organising pneumonia: 'Has there been a widespread failure to identify and treat this prevalent condition in COVID-19?'. BMJ Open Respir Res.

[8] Udwadia ZF, Koul PA, Richeldi L (2021). Post-COVID lung fibrosis: the tsunami that will follow the earthquake. Lung India.

[9] Akhtar A, Ul Abideen Z (2015). Acute fibrinous and organizing pneumonia masquerading as a lower respiratory tract infection: a case report and review of the literature. BMC Res Notes.

[10] Navas-Blanco JR, Dudaryk R (2020). Management of respiratory distress syndrome due to COVID-19 infection. BMC Anesthesiol.

